# Analyst optimism, information disclosure, and stock price collapse risk: Empirical insights from China’s A-share market

**DOI:** 10.1371/journal.pone.0297055

**Published:** 2024-03-28

**Authors:** Yang Li, Yingchun Zhang, Rui Ma, Ruixuan Wang

**Affiliations:** 1 School of Economic and Management, Qinghai Minzu University, Xining, Qinghai, P. R. China; 2 Business School, Qinghai University of Science and Technology, Xining, Qinghai, P. R. China; 3 Qinghai Provincial Key Laboratory of Big Data in Finance and Artificial Intelligence Application Technology, Xining, Qinghai, P. R. China; Bahauddin Zakariya University, PAKISTAN

## Abstract

This study selects stock data of listed companies in China’s A-share stock market from 2011 to 2020 as research samples. Using a fixed-effects model, it examines the impact of analyst optimism on stock price collapses and the moderating effect of information disclosure quality. Simultaneously, it conducts additional research to explore the potential transmission mechanisms involved. The main findings are as follows: Firstly, a positive correlation exists between analyst optimism and the risk of stock price collapse. Secondly, improving information disclosure quality of listed companies can enhance the positive impact of analyst optimism on the risk of stock price collapses and expedite the market’s adjustment of overly optimistic valuations of listed companies. Additionally, analyst optimism can increase the risk of stock price collapses by affecting institutional ownership. These findings provide theoretical support for regulatory authorities to revise and improve the "information disclosure evaluation" system, regulate the analyst industry, guide analyst behavior, and encourage listed companies to enhance internal governance and improve information disclosure practices.

## 1. Introduction

The stability of the capital market plays a crucial role in promoting the robust development of a nation and its overall economic system. It holds immense significance in promoting economic growth, optimizing resource allocation, strengthening investor confidence, and ensuring the stability and growth of the economy. The stability of the capital market depends on various factors, and one important factor is the occurrence of stock price collapse, defined as rapid and significant declines in stock prices within a short period [1]. Historically, stock price collapses have resulted in substantial instability in the capital market. For instance, the stock market crash in China in 2015 witnessed stocks with greater crash susceptibility experiencing more severe declines than the overall market [2]. The global stock market crash in March 2020, linked to the COVID-19 pandemic, exceeded the volatility of the Great Depression, the Great Financial Crisis, and the Spanish Flu pandemic, significantly impacting the stability of the capital market [3]. The collapse of Silicon Valley Bank (SVB) in the United States in 2023 also had extensive implications for global stock markets, leading to disruptions in the financial system [4].

During these historically significant financial crisis events, numerous instances occurred where individual stocks saw a sharp decline in their prices. Such significant drops in individual stock prices can lead to significant losses for all participants in the capital market, harming investors’ interests, eroding their confidence, and impeding the healthy development of the capital market. Besides stock price collapses triggered by systemic risks, specific behaviors of listed companies can also contribute to a significant decline in stock prices. For example, insufficient internal control measures within the company enabled company executives to embezzle corporate assets; companies violated regulations by engaging in unauthorized information disclosure and faced regulatory sanctions from the relevant authorities [5, 6]. Concerning the underlying causes, the prevailing view suggests that information asymmetry is the main driving force behind these incidents [7, 8].

As China’s securities market undergoes ongoing and deepening reforms, the analyst industry is experiencing continuous development and growth. Serving as intermediaries of information, analysts primarily collect data on listed companies and disseminate it to small and medium-sized investors in the market [6]. They achieve this through activities like publishing research reports to assess the value of listed companies’ securities and developing appropriate investment strategies for clients. These practices have long been recognized as methods to improve transparency in listed companies and reduce information imbalances. However, in the real securities market, analysts may issue biased ratings due to the influence of their interests. This can result in cases where analysts issue optimistic ratings, but the stock prices of listed companies subsequently decline. Additionally, the listed companies themselves, as the direct producers of operating information, can significantly affect the quality of analysts’ research reports through the accuracy and comprehensiveness of the information they disclose to the market.

Therefore, it is of significant practical importance to examine the impact of analyst optimism on the risk of stock price collapse and the role of information disclosure in this relationship. Based on the existing literature, it can be observed that current research predominantly focuses on studying stock price collapse from the perspective of internal company characteristics or market factors. Some scholars have separately conducted empirical research from the perspectives of securities analysts and information disclosure quality. There is limited research that combines analyst ratings and the quality of corporate information disclosure to study their joint impact on the risk of stock price collapse. Furthermore, unlike previous research, this paper explores the moderating effect of information disclosure quality through an investigation of the interaction between analyst optimism and information disclosure quality. To broaden the scope of this article, institutional ownership is introduced as a mediating variable to study the transmission mechanism, and empirical analysis confirms the existence of the institutional ownership transmission channel. This diversification enriches the understanding of factors influencing stock price collapse and possible transmission mechanisms. In conclusion, this study significantly enriches existing theoretical research. It refines the investigation of factors influencing stock price collapse, offering insights for regulators into more specialized areas. Furthermore, it expands upon the current literature on stock price collapse risk, introducing an innovative research perspective.

To address the research gap identified above, this study initiates by reviewing and analyzing pertinent literature and summarizing it through a comprehensive literature review. Subsequently, by analyzing the relationships and mechanisms involving analyst optimism, information disclosure quality, institutional ownership, and stock price collapse, the study formulates research hypotheses. Next, the research employs a sample of Chinese A-share listed companies from 2011 to 2020 to investigate the relationship between analyst optimism and the risk of stock price collapse from two dimensions: the negative skewness coefficient (ncskew) and the volatility ratio (duvol). It also examines whether information disclosure quality acts as a moderator in this relationship. Furthermore, to enrich the study, institutional ownership is introduced to supplement the investigation of the transmission mechanism in the aforementioned relationships. The study primarily employs a "two-way fixed-effects model," a commonly used method in panel data analysis, particularly suited for cases with both individual and time fixed effects. Given that the study involves data from multiple time points and companies, this model allows for the control of company-specific fixed effects, helping to mitigate the potential impact of unobservable factors within the companies. This control is crucial for investigating the relationship between analyst optimism and the risk of stock price collapse. Additionally, the study introduces "industry" and "year" as control variables to enable a more precise examination of the relationships between analyst optimism, information disclosure quality, institutional ownership, and the risk of stock price collapse, accounting for potential confounding factors and enhancing the study’s credibility. In the final part of this article, based on the empirical findings, recommendations are provided for policymakers, listed companies, and investors. These recommendations offer theoretical guidance for future research in this field.

## 2. Literature review

### 2.1 Analyst optimism

Analyst optimism refers to the tendency of financial analysts to issue earnings forecasts or stock recommendations that are more positive than warranted by objective economic conditions or firm performance [9, 10]. This phenomenon has been studied extensively in the literature, with various causes, consequences, and mitigating strategies proposed. Meanwhile, the academic community holds both positive and negative perspectives on analyst optimism.

Considering the positive impact perspective. Analyst optimism can play a significant role in shaping investor behavior, company performance, and market dynamics. From the perspective of individual investors, analyst optimism has the potential to influence the emotions and investment decisions of individuals, consequently impacting stock market returns. Limongi Concetto and Ravazzolo (2019) discovered that, in the U.S. market, the sentiment index reflecting analysts’ optimistic sentiment accurately predicts stock returns for the next month, outperforming the random walk benchmark. This suggests that analysts’ optimistic sentiment can guide investors to make more informed decisions, potentially resulting in improved investment outcomes [11]. According to Ciccone (2003) and the research findings of Chang and Choi (2017), analysts’ optimistic sentiment helps stimulate investor interest, consequently driving stock prices upward [12, 13]. Chhatwani and Mishra (2021), in their study of the correlation between financial fragility and financial optimism, found that analyst optimism can assist investors in maintaining optimistic financial expectations during economic crises, thereby stabilizing the market and contributing to economic recovery [14]. The innovation capability and efficiency of a company significantly impact its competitive advantage [15]. From the perspective of the operations of a publicly listed company, analyst optimism can encourage innovation and the pursuit of sustainable innovative practices, contributing to long-term performance [16]. Simultaneously, these optimistic analyses can create pressure on management to meet or exceed these expectations, leading to more aggressive business strategies and innovation [17]. This can improve company performance and boost stock prices, benefiting investors who rely on analyst predictions for investment decisions. Additionally, analysts’ optimistic sentiment can result in a more positive assessment of company risks, encouraging investment in companies perceived as having growth potential. This can increase the capital availability for these companies, enabling them to invest in new projects and expand their operations [18]. Moreover, analyst optimism can have a spillover effect between markets.

Furthermore, critics of analyst optimism contend that when conflicts of interest are present, analysts tend to provide more positive ratings for listed companies due to various reasons. This situation can lead to an upward adjustment of stock ratings, which in turn raises the risk of a stock price collapse and hinders the healthy development of the securities market. Lehmer et al. (2022) studied the relationship between analyst optimism and the trading volume of brokerage firms. They found that analysts may be incentivized to issue optimistic ratings to increase the trading volume of brokerage firms [19]. Irvine (2004) noted a substantial rise in trading volume and commission revenue for securities firms within two weeks following analysts issuing buy recommendations [20]. In a study focused on stock pledged repo transactions, Zhang et al. (2020) found that affiliated analysts, as compared to non-affiliated analysts, tend to release more optimistic stock recommendations [21]. The above research confirms that the forecasts published by analysts will ultimately impact their own income. Therefore, analysts are more inclined to release positive stock recommendations to the market rather than providing objective investment advice based on analysis. Furthermore, Cremers et al. (2021) suggest that there is evidence of return predictability based on analyst recommendations and fund turnover, indicating potential herding behavior among financial analysts [22]. Barber and Odean (2000) investigated analysts’ herding behavior and discovered that analysts frequently conform to the prevailing consensus when issuing ratings, possibly to avoid deviating from market expectations and to preserve their reputation. Consequently, this conformity can lead to the publication of optimistic ratings similar to other analysts [23]. Xu et al. (2013) discovered a positive correlation between an increase in analysts’ attention to a company and an elevated risk of stock price collapse. Importantly, by categorizing analysts into optimistic and non-optimistic groups for research purposes, it was found that the positive correlation only exists among optimistic analysts [24].

In summary, analyst optimism, characterized by positive forecasts or recommendations beyond objective conditions, is extensively studied with diverse perspectives. Positive views emphasize its impact on investor behavior, market dynamics, and company performance, suggesting that analyst optimism stimulates the stock market, encourages innovation, and contributes to economic recovery. However, critics argue that conflicts of interest may lead to overly positive ratings, potentially affecting market health and increasing the risk of stock price collapse.

### 2.2 Disclosure of information

Information disclosure plays a vital role in the securities market, exerting a significant impact on investor decision-making and market efficiency [25]. Presently, the academic community generally believes that the level of information disclosure is closely linked to the risk of stock price collapse. Jin et al. (2022), by studying the relationship between epidemics and subsequent quarterly stock price collapse risk, found that companies’ voluntary disclosure of adverse events could result in a loss of investor confidence, thereby increasing the likelihood of stock price collapse [26]. Conversely, Chamley and Gale (1994), Madhani (2008), Dierker and Subrahmanyam (2017) argue that inadequate information disclosure can increase the risk of stock price collapse because information asymmetry may lead to excessive or delayed market reactions [27], amplifying market and investor uncertainty [28] and affecting investors’ ability to make accurate decisions [29]. Besides, in the research on Corporate Social Responsibility (CSR) of Chinese companies, Dai et al. (2019) found an inverted U-shaped nonlinear relationship between the level of information disclosure and the risk of stock price collapse [30]. This suggests that although the initial increase in information disclosure may worsen the risk of stock price collapse, further disclosure may ultimately mitigate this risk. Moreover, the extent of information disclosure plays a crucial role in safeguarding investor interests. Adequate disclosure empowers investors to gain a better understanding of a company’s business status, risks, and prospects, enabling them to make informed investment decisions [29]. Inadequate disclosure of information can result in investors misjudging risks and prospects, thereby increasing the risks of investment losses and stock price collapse [31]. Consequently, the impact of information disclosure quality on different participants in the capital market is primarily manifested in two aspects: First, the improvement of information disclosure quality can enhance the accuracy of analysts’ research reports. Second, the improvement of information disclosure quality can significantly restrain managerial self-interest behavior.

In terms of enhancing the accuracy of analyst research reports, Byard and Shaw (2003) revealed a significant influence of information disclosure quality on the accuracy of analyst forecasts for listed companies [32]. This finding was further supported by Fang’s research in 2007, which specifically demonstrated that higher information disclosure quality by listed companies provides analysts with more effective information, reducing their reliance on accounting earnings data and thereby improving the accuracy of their forecasts [33]. Empirical research conducted by Li and Xiao in 2015 found that when company management provides performance forecasts, market participants gain additional insights into the company’s expected earnings. This enables analysts to make more precise earnings predictions and helps alleviate information asymmetry in the securities market [34]. Building on the aforementioned research, Wang et al. (2017) emphasized that this impact varies based on company characteristics, such as ownership structure, profit quality, and corporate governance [35]. Furthermore, Hu et al. (2016) illustrates that when listed companies utilize internet platforms and other channels to disseminate information to a wider range of participants, securities analysts gain access to more information, thereby mitigating information asymmetry to some extent and reducing the likelihood of stock price collapse for listed companies [36]. Tseng and Shih (2022) compared the effects of accountant assurance with those of other third-party assurance and found that accountant assurance had a more significant impact on increasing the accuracy of analyst forecasts and reducing the dispersion of analyst forecasts compared to other third-party assurance providers [37]. However, it’s important to note that while enhanced disclosure quality can improve forecast accuracy, it is not the sole factor at play. Analysts’ individual expertise, experience, and the tools they use to interpret information also play crucial roles. Additionally, external factors such as political, economic, social, technological, legal, and environmental factors can all potentially influence the accuracy of forecasts and market efficiency [38].

Regarding the restraint of management’s self-serving behavior, Li and Myers (2006) [39] propose that the advantage of having internal information enables them to be the first to receive negative news that could have an adverse impact on stock prices. However, due to personal interests, management often conceals such negative information. As time passes, when these accumulated negative messages surpass a certain threshold, they eventually reach the market, eroding investor confidence. Moreover, Bleck and Liu (2007) discovered that companies with lower information transparency exhibit a higher frequency of profiting from information asymmetry compared to those with higher transparency, as they may opt to invest in projects with negative net present value that benefit themselves. They employ tactics such as concealing information to temporarily mask actions that harm the investors. However, when the losses from these projects become significant, it can trigger a collapse in stock prices. In contrast, companies with higher information transparency experience a significant reduction in the occurrence of such phenomena, thereby diminishing the risk of stock price collapse [40].

In conclusion, information disclosure is crucial in the securities market, significantly influencing investor decisions and market efficiency. Academic consensus asserts that the level of disclosure is closely linked to the risk of stock price collapse, and improving information disclosure quality enhances the objectivity and accuracy of analyst ratings while also restraining self-serving behaviors of management.

### 2.3 Comprehensive review of literature

Through the review of existing literature, it can be observed that when analysts can effectively fulfill their roles and provide oversight of listed company behavior, the frequency of Earnings Management (the act of manipulating and adjusting the reported accounting income information to maximize the interests of the entity while adhering to accounting standards) decreases. Simultaneously, the difficulty of concealing various negative information generated in corporate operations also increases, thereby mitigating the risk of stock price collapse for listed companies. Conversely, when analysts are influenced by various forces or their interests are tied to listed companies and institutional investors, thus compromising their objectivity and independence, the optimistic estimates they release fail to truly reflect the actual operating conditions of the company. Consequently, information is not effectively transmitted to market investors in a timely manner, while the degree of information asymmetry deepens, thereby increasing the risk of stock price collapse for listed companies.

The prevailing viewpoint on the information disclosure of listed companies suggests that a low level of information disclosure increases the risk of stock price collapse. Additionally, the quality of information disclosure plays a crucial role in the capital market. It enhances the accuracy of analyst research reports by providing analysts with more effective information, reducing reliance on accounting earnings data, and enhancing forecast precision. Furthermore, it acts as a restraint on management’s self-serving behavior by reducing information asymmetry, promoting transparency, and mitigating the likelihood of stock price collapse. These findings emphasize the importance of improving information disclosure practices in enhancing market efficiency and investor confidence.

Existing literature on stock price collapse predominantly focuses on internal company characteristics or market factors. However, there are research gaps in this field. Firstly, there is limited research that investigates analyst ratings and information disclosure quality as independent entities. Studies on stock price collapse risk often overlook the potential interaction between these two crucial elements, which may play an interactive role in risk assessment. Secondly, there is a need for in-depth research on the impact of institutional holdings on stock price collapse risk. Existing studies on the comprehensive effects of institutional holdings are relatively limited, and institutional investors, as significant market participants, may have a substantial moderating effect on stock price collapse. Furthermore, the existing literature does not adequately consider other potential factors influencing stock price collapse, such as market sentiment, regulatory changes, etc. Therefore, it is crucial to conduct more comprehensive and integrated research to uncover the synergistic effects among factors such as analyst ratings, information disclosure quality, institutional holdings, and others. This approach aims to provide a more holistic understanding and assessment of stock price collapse risk.

In the current context of the ongoing shift in the focus of China’s economic development, it is crucial to effectively communicate company information to investors, enabling the full exploration of the commercial value of promising companies and maximizing the role of the financing platform. Simultaneously, ensuring the stability of the capital market and providing a favorable environment for all market participants are essential prerequisites for China’s high-quality economic development. Therefore, conducting comprehensive research on the relationship between analyst optimism, information disclosure, and the risk of stock price collapse, and elucidating their inherent connections, can significantly enhance investor protection, improve market efficiency, reduce information asymmetry, and provide robust support to regulators and policymakers. Ultimately, this research can contribute to the stability and development of the capital market in China.

## 3. Theoretical analysis and research hypotheses

### 3.1 The relationship between analyst optimism and stock price collapse risk

With the ongoing reforms and advancements in China’s securities market, including the establishment of the Science and Technology Innovation Board (STAR Market) and the Beijing Stock Exchange, coupled with the growing number of listed companies, the market has witnessed a surge in diverse and intricate information. Consequently, the role of securities analysts in the market has gained increasing significance as they act as intermediaries bridging the gap between listed companies and investors, with the goal of minimizing information asymmetry [41]. However, due to the relatively delayed inception of China’s securities market, it still lags behind Western markets in terms of maturity. Existing literature has analyzed that Chinese securities analysts often tend to issue optimistic ratings on listed companies for various reasons when participating in the stock market [42–44]. In the Chinese market, securities analysts unavoidably establish business connections with institutional investors and listed companies. Within these business interactions, situations arise where all parties engage in "mutually beneficial" exchanges, leveraging their respective advantages [43, 45, 46]. For example, when analysts release positive research reports about a listed company, it can attract investors to purchase its stock. During such instances, institutional investors and company executives may capitalize on the opportunity to sell and exit the market, securing excess profits. In return, analysts can gain access to more privileged information about listed companies in the future, as well as expedited promotion opportunities.

However, if these optimistic estimates prove to be unfounded or overly optimistic, they can create an expectation gap between market expectations and actual company performance. If the company fails to meet the optimistic projections, it can result in a significant correction or even a collapse in the stock price [47]. This is particularly true when investors have based their investment decisions on the optimistic estimates, inflating the stock price beyond its intrinsic value. Additionally, the phenomenon of herding behavior among investors can further exacerbate the risk of stock price collapse [48]. When a majority of analysts provide optimistic estimates for a specific stock, it can create a positive feedback loop, prompting investors to follow the herd and invest in the stock without conducting thorough independent analysis. This can lead to an overvaluation of the stock and increase vulnerability to a sharp decline if the optimistic estimates turn out to be inaccurate. Therefore, the relationship between analysts’ optimistic estimates and the risk of stock price collapse is intricate. Considering the theoretical analysis from both perspectives, while optimistic estimates can fuel market optimism and drive stock prices higher in the short term, they also introduce the potential for misaligned expectations and increased vulnerability to price corrections, thereby heightening the risk of stock price collapse. Therefore, this article proposes the following hypotheses:

Hypothesis 1a: Analyst optimism can exacerbate the risk of stock price collapse.

Furthermore, due to the complexity of the impact and transmission mechanisms of analyst optimism on stock price collapses, this study incorporates institutional ownership as an intermediary variable to enhance the research on transmission mechanisms.

Institutional investors play a crucial role in the Chinese securities market. Empirical analysis conducted by An and Zhang (2013) on mature capital markets in the West revealed a significant negative correlation between institutional ownership and the risk of stock price collapses [49]. However, in the current stage of the Chinese capital market, factors such as limited management experience and inadequate regulatory frameworks contribute to situations where institutional investors and securities analysts align their interests through agency relationships and economic incentives. In their agency relationships, institutional investors often encounter constraints in accessing complete information about listed companies due to cost limitations. Conversely, analysts can leverage their professional expertise and the support of brokerage firms to access superior information channels compared to institutional investors, thereby providing investment advice to the latter. From an economic standpoint, the substantial funds involved in institutional investors’ trading activities generate significant commissions for securities firms. Consequently, some securities analysts may issue optimistic ratings to mask the selling of stocks held by institutional investors. Furthermore, empirical research by Jiang and Kim (2015) demonstrates that Chinese institutional investors have a short holding period, engage in frequent stock trading, and exhibit significant short-term speculative behavior [50]. These factors indicate that higher institutional ownership is associated with an increased likelihood of future stock price collapses. Additionally, as institutional ownership rises, it leads to short-term stock price increases and creates a demand for value regression in the future. Drawing on these considerations, the following hypotheses are proposed in this article:

Hypothesis 1b: Higher levels of institutional ownership heighten the risk of stock price collapses through the form of optimistic optimism.

### 3.2 The regulatory effect of information disclosure quality

Gentzkow (2016) conducted empirical research and identified a correlation between a company’s profitability and the quality of its information disclosure. Companies with higher profitability tend to highlight their favorable financial performance to the market, attracting increased social attention and aiming for stock price appreciation. Consequently, these companies are more inclined to provide a greater amount of information [51]. In essence, when the information disclosure quality of listed companies improves, they usually disseminate more positive information to the market.

Meanwhile, the ratings assigned by analysts significantly influence investors’ decisions and market dynamics. However, studies indicate that analysts have certain preferences during the rating process, indicating that they tend to assign higher ratings to specific stocks or companies based on various factors. It is challenging for them to achieve complete objectivity and fairness when providing ratings. Additionally, Bourveau et al. (2022) suggests that higher information disclosure quality enables analysts to assess a company’s performance and prospects more comprehensively and accurately, leading them to assign higher ratings to such companies [52]. Research conducted by Fang et al. (2022) highlights that well-known companies with a strong market position often receive significant attention and importance from analysts. Analysts may be more inclined to assign higher ratings to these companies in order to safeguard their reputation and maintain a positive cooperation relationship [53].

The mentioned research suggests that higher information disclosure quality may lead analysts to adopt a more optimistic outlook on a company’s prospects and value. However, if this optimistic analysis fails to sufficiently consider potential risks and issues, it can increase the risk of stock price collapse. In other words, when the information disclosure quality of listed companies improves, it amplifies the impact of analysts’ optimism, potentially leading to an underestimation of potential risks and an increased likelihood of stock price collapse. The analysis suggests that the influence of information disclosure quality and analyst ratings on the risk of stock price collapse is positively moderated, indicating an interaction between the two. The enhancement of information disclosure quality strengthens the exacerbating effect of analyst ratings on the risk of stock price collapse. Consequently, this paper proposes the following hypothesis:

Hypothesis 2a: The improvement of information disclosure quality of listed companies contributes to strengthening the exacerbating effect of analyst optimism on the risk of stock price collapse.

Additionally, Research findings have established that information asymmetry is an important factor contributing to the collapse of stock prices [7, 8]. This asymmetry primarily arises from the fact that the management of listed companies and institutional investors gain early access to crucial information regarding corporate decisions. On the contrary, ordinary investors in the market face delays in obtaining the same information. Consequently, the management and institutional investors can exploit this advantage to profit in the securities market, thereby amplifying the risk of stock price collapse. To address this risk resulting from information asymmetry, enhancing the quality of information disclosure by listed companies unquestionably emerges as the most effective solution. To ensure the integrity of information disclosure by companies listed on the A-share market in China, a system known as the "information disclosure evaluation system" has been implemented. This system involves regular or irregular assessments and reviews of the information disclosure practices of listed companies by regulatory bodies such as the China Securities Regulatory Commission and the stock exchanges including the Shanghai Stock Exchange and the Shenzhen Stock Exchange. These evaluations are based on criteria that encompass the accuracy, timeliness, comprehensiveness, quality, and transparency of the disclosed information. The evaluation results are used to provide corresponding ratings or assessments.

Based on the previous analysis, if analyst optimism is positively associated with the risk of stock price collapse, it suggests that the A-share market, which adheres to various evaluation standards, has a mechanism in place to automatically rectify excessively optimistic expectations of analysts by reducing stock prices or even triggering market collapses. If the "information disclosure evaluation" system can effectively regulate the quality of information disclosure by listed companies, the enhancement of information disclosure quality should facilitate the self-correcting mechanism of market expectations, thereby expediting the rational correction of stock prices for companies that have been overly valued due to excessive optimism. Consequently, this paper proposes the following hypothesis:

Hypothesis 2b: The improvement in the quality of information disclosure by listed companies can accelerate the rational regression of overly optimistic valuations of listed companies in the market.

## 4. Research design

### 4.1 Data source and sample selection

The data for this study is obtained from the Wind database and CSMAR (China Stock Market & Accounting Research) database. This study selects China’s A-share listed companies from 2011 to 2021 as the research sample. The original data is processed as follows:

1. Exclusion of listed companies in the financial sector.
2. Exclusion of samples with missing data.
3. Exclusion of ST and *ST (common stock symbols used in the Chinese A-share market to indicate potential risks and issues, requiring caution from investors) listed companies.

After the processing, a total of 18,010 observations are obtained. To mitigate the influence of extreme values on the results, trimming treatment is applied to continuous variables at the 1st and 99th percentiles. The data processing is performed using Stata 17 software.

### 4.2 Variable design

#### 4.2.1 Dependent variables

Building on the comprehensive studies of Wang, Cao and Ye (2015), Kim, Li, and Zhang (2011), Xu et al. (2014), Kim and Zhang (2014, 2016) [54–59]. In this paper, the risk of stock price collapse is assessed using Ncskew (the coefficient of negative return skewness) and Duvol (the stock price volatility ratio of weekly returns). The specific methodology is outlined below:

In the first step, regression is conducted using the Model 1:

$$R_{i,t}=\partial_{i}+\beta_{1}R_{m,t-2}+\beta_{2}R_{m,t-1}+\beta_{3}R_{m,t}+\beta_{4}R_{m,t+1}+\beta_{5}R_{m,t+2}+\varepsilon_{i,t}$$ (1)

In the equation *R*_*i*,*t*_ represents the stock return of stock *i* in week *t* (considering the reinvestment of cash dividends) *R*_*m*,*t*_ denotes the market return in week *t* (calculated after considering market capitalization weighting). Moreover, to account for the impact of stock asynchronous trading, the equation includes leading and lagging terms of the market return.

In the second step, based on the regression results of *ε*_*i*,*t*_, the stock-specific weekly return is calculated using Model 2:

$$W_{i,t}=ln\;(1+\varepsilon_{i,t})$$ (2)

In the third step, the Ncskew and the Duvol are calculated using *W*_*i*,*t*_, as shown in Model 3 and 4:

$$N{cskew}_{1,t}=-[n*(\text{n}-1)*3/2*\sum W_{i,t3}]/[(n-1)*(n-2)*(\sum W_{i,t2})*3/2]$$ (3)

Where *n* represents the number of trading weeks in a year for stock *I*, a larger value of Ncskew indicates a higher risk of stock price collapse.


$$D{uvol}_{1,t}=ln\{[(n_{u}-1)(\sum_{down}W_{i,t}^{2})]/[(n_{d}-1)(\sum_{up}W_{i,t}^{2})]\}$$ (4)

Where *n*_*u*_ represents the number of weeks in which the weekly return of stock *i* is greater than the annual average return *W*_*i*,*t*_. Besides, *n*_*d*_ represents the number of weeks in which the weekly return of stock *i* is lower than the annual average return *W*_*i*,*t*_.

#### 4.2.2 Explanatory variables

In line with the study conducted by Li, Wu and Gao (2018) [60], this research incorporates analyst ratings as explanatory variables. The data for these ratings is sourced from the CSMAR database, where ratings provided by analysts from different research institutions are standardized on a scale of 1 to 5 (1 represents "Sell", 2 represents "Reduce", 3 represents "Neutral", 4 represents "Hold" and 5 represents "Buy"), with the highest score representing the most positive rating. For this study, the annual average rating is chosen as the representative measure.

#### 4.2.3 Moderating variables

In this study, the quality of information disclosure by listed companies serves as a moderating variable. It is assessed through an evaluation conducted by the Shenzhen Stock Exchange, which evaluates the timeliness, accuracy, completeness, and legality of the information disclosed by listed companies. The quality of information disclosure is classified into four levels: excellent, good, qualified, and unqualified, with corresponding values of 4, 3, 2, and 1, respectively.

#### 4.2.4 Mediating variables

To enhance the understanding of the transmission mechanism of stock price collapse risk, institutional ownership is used as a mediating variable. Institutional ownership is measured by the percentage of shares held by institutional investors at the end of the year for listed companies, with data sourced from the CSMAR database.

#### 4.2.5 Control variables

To comprehensively analyze the impact of explanatory variables on the dependent variable, this study includes the following indicators as the main control variables, drawing on previous literature (Kim et al., 2011; Xu et al., 2012) [44, 56]:

1. Debt-to-asset ratio (Lev): Ratio of total liabilities to total assets. A higher value suggests a greater level of debt, indicating relatively higher financial risk.
2. Firm size (Size): The natural logarithm of the total assets of the listed company at the end of the period.
3. Board size (Abord): The natural logarithm of the number of board members minus the number of independent directors.
4. Liquidity ratio (Flu): The ratio of current assets to current liabilities at the end of the year.
5. Largest shareholder ownership (Cr1): The proportion of shares held by the largest shareholder in the listed company during the year. A higher proportion indicates a higher risk of stock price collapse.
6. Company age (Age): The natural logarithm of the number of years since the company’s establishment.

Additionally, in the subsequent empirical analysis of this study, controls were introduced for individual effects and year effects. Variable definitions are shown in Table 1.

**Table 1:** Variable definition.

Type	Name	Symbol	Definition
Dependent variables	Coefficient of negative return skewness	Ncskew	Computed using formula 3, measures the risk of stock price collapse.
	Stock price volatility ratio	Duvol	Computed using formula 4, measures the risk of stock price collapse.
Explanator variable	Analyst ratings	Analyst	Standardized ratings of analyst investment recommendations on a scale of 1 to 5 from low to high
Moderating variable	Information disclosure score	Score	Standardized ratings of information disclosure quality for listed companies on a scale of 1 to 4 from low to high.
Mediating variable	Institutional ownership	Institution	Percentage of shares held by institutional investors in listed companies at the end of the year.
Control variable	Debt-to-asset ratio	Lev	Ratio of total liabilities to total assets.
	Firm size	Size	Natural logarithm of total assets of the listed company at the end of the period.
	Board size	Abord	Natural logarithm of the number of board members minus the number of independent directors.
	Liquidity ratio	Flu	Ratio of current assets to current liabilities at the end of the year.
	Largest shareholder Ownership	Cr1	Proportion of shares held by the largest shareholder in the listed company during the year.
	Company age	Age	Natural logarithm of the number of years since the company’s establishment.
Dummy Variable	Ownership type	State	1 indicates state-owned enterprises, 0 otherwise.

### 4.3 Model construction

This study primarily employs a two-way fixed effects model, which offers several advantages [61–63]. Firstly, the methodology incorporates individual and time fixed effects to control for unobserved factors that may influence the regression results, reducing omitted variable bias and enhancing result accuracy. Secondly, it helps control for individual and time heterogeneity, ensuring a more accurate analysis of the impact of explanatory variables on the dependent variable without attributing effects to individual or time differences. Thirdly, the two-way fixed effects model reduces estimate variance, enhancing regression model efficiency. It also addresses endogeneity issues under fixed effects, leading to more consistent estimation results. Lastly, the methodology is well-suited for panel data analysis, where observational units have multiple observations across different time periods or locations, allowing it to capture variability and dynamic features effectively.

#### 4.3.1 Testing model for analyst optimism and stock price collapse risk

To examine hypothesis 1a, which investigates the association between analyst optimism and stock price collapse risk, this study establishes a multiple regression model (Model 5) as follows:

$$\text{Risk}=\beta_{0}+\beta_{1}*Analyst+\sum \beta_{i}*Control_{i}+\sum Year+\varepsilon$$ (5)

The evaluation model for the relationship between analyst optimism and stock price collapse risk is based on the measurements of coefficient of negative return skewness (Ncskew) and stock price volatility ratio of weekly returns (Duvol) as indicators of stock price collapse risk (Crashrisk). The model includes control variables (Control_i_) such as debt-to-asset ratio (Lev), firm size (Size), board size (Abord), liquidity ratio (Flu), largest shareholder ownership (Cr1), and company age (age). Model 5 primarily examines the coefficient *β*_1_ associated with analyst ratings (Analyst). If this coefficient is significantly positive, it indicates a significant positive correlation between analyst ratings and the risk of stock price collapse. In other words, the higher the optimism level of analyst ratings for a listed company, the higher the likelihood of stock price collapse for that company.

#### 4.3.2 Testing model for the mediating effect of institutional ownership

To test hypothesis 1b, this study adopts the mediation analysis approach proposed by Jiang (2022) [64]. Building upon the earlier theoretical foundation, it is suggested that institutional ownership is one of the mechanisms through which analyst optimism influence stock price collapse risk. Institutional ownership directly impacts stock price collapse risk due to the selling pressure resulting from increased institutional holdings and the value regression demand of the company’s stock price, thereby raising the risk of stock price collapse. Therefore, after conducting the regression of the dependent variable on the independent variable in Model 1, the following model is employed to examine the causal relationship between the mediating variable and the independent variable:

$$Institution=\alpha_{0}+\alpha_{1}*Analyst+\sum \alpha_{i}*Control_{i}+\sum Year+\varepsilon$$ (6)

The significance of the coefficient *β*_1_ in Model 5 and the coefficient *α*_1_ in Model 6 are sequentially tested to demonstrate whether institutional ownership acts as a mediator.

The aforementioned mediation analysis can assist researchers in understanding the causal relationship between the independent and dependent variables. By examining the presence and role of the mediating variable, researchers can determine whether the impact of the independent variable on the dependent variable occurs through the mediating variable. Moreover, mediation analysis provides a more accurate explanation, revealing the mechanisms behind the relationship between the independent and dependent variables. It helps us understand how the independent variable influences the dependent variable through the mediating variable, thereby offering a more comprehensive interpretation of the study results.

#### 4.3.3 Testing model for the moderating effect of information disclosure quality

To validate hypothesis 2, the following multiple regression model (Model 7) is designed to examine the impact of information disclosure quality on the relationship between analyst optimism and stock price collapse risk:

$$\text{Risk}=\beta_{0}+\beta_{1}*A\text{nalyst}+\beta_{2}*S\text{cor}e+\beta_{3}*Analyst*S\text{core}+\sum \beta_{i}*Control_{i}+\sum Year+\varepsilon$$ (7)

In light of the research, as the interaction term in Model 7 comprises the product of analyst ratings (*Analyst*) and information disclosure quality (*Score*), this study centers the interaction term in Model 7 to enhance the interpretability of the coefficient linked with it. If the coefficient *β*_3_ related to the interaction term shows significant positivity in the empirical results, it signifies that information disclosure quality amplifies the positive correlation between analyst ratings and the risk of stock price collapse.

## 5. Empirical study

### 5.1 Descriptive statistics

Descriptive statistics analysis was conducted using Stata 17 to examine the relevant data in this study. The analysis results include the mean, standard deviation, minimum, and maximum values of each variable. The detailed results are presented in Table 2:

**Table 2:** Descriptive statistics of variables.

Variable	Sample Size	Mean Value	Standard Deviation	Minimum Value	Maximum Value
**Ncskew**	18010	-0.283	0.728	-5.171	4.129
**Duvol**	18010	-0.186	0.485	-2.223	2.239
**Analyst**	18010	4.384	0.433	2.000	5.000
**Score**	18010	3.146	0.611	1.000	4.000
**Institution**	18010	0.400	0.241	0.000	1.870
**Size**	18010	22.385	1.336	19.087	28.636
**Lev**	18010	0.407	0.199	0.008	1.252
**Abord**	18010	2.126	0.196	1.099	2.890
**Flu**	18010	2.716	4.126	0.106	190.869
**CR1**	18010	0.344	0.149	0.024	0.900
**Age**	18010	2.870	0.354	1.099	4.159

Based on the information presented in Table 2, the mean values of the Ncskew and Duvol indicators, which measure stock price collapse risk, are -0.283 and -0.186 respectively. Their standard deviations are 0.728 and 0.485. The maximum value of Ncskew is 4.129, while the minimum value is -5.171. As for Duvol, its maximum value is 2.239 and the minimum value is -2.223. These findings suggest notable variations in stock price collapse risk across different listed companies.

Analyst ratings (Analyst) for sampled Chinese listed companies range from 2.000 to 5.000, with an average of 4.384, indicating an overall optimistic trend among Chinese analysts. The average information disclosure quality score (Score) is 3.146, suggesting high disclosure standards, but notable variations exist between companies. Institutional ownership (Institution) averages 0.4, revealing a lower proportion compared to developed markets, with significant differences among companies. Debt-to-asset ratios (Lev) vary widely, with an average of 0.407, reflecting diverse debt structures. Liquidity ratios (Flu) range from 0.106 to 190.869, emphasizing considerable disparities in the debt-servicing capacities of listed companies in China.

### 5.2 Correlation analysis

In this study, the Pearson correlation coefficient method is used to examine the relationships between analyst optimism, information disclosure quality, institutional ownership, and the relationships between control variables and stock price collapse risk. The correlation coefficients are presented in Table 3.

**Table 3:** Correlation analysis of variables.

	**Ncskew**	**Duvol**	**Analyst**	**Score**	**Institution**	**Size**	**Lev**	**Abord**	**Flu**	**CR1**	**Age**
**Ncskew**	1.000										
**Duvol**	0.864***	1.000									
**Analyst**	0.002	0.015**	1.000								
**Score**	-0.026***	-0.025***	-0.094***	1.000							
**Institution**	0.009	-0.007	-0.067***	0.198***	1.000						
**Size**	-0.051***	-0.081***	-0.151***	0.195***	0.467***	1.000					
**Lev**	-0.057***	-0.072***	-0.041***	-0.070***	0.243***	0.571***	1.000				
**Abord**	-0.019***	-0.022***	0.044***	0.076***	0.197***	0.233***	0.139***	1.000			
**Flu**	0.031***	0.041***	0.077***	0.025***	-0.138***	-0.280***	-0.476***	-0.093***	1.000		
**CR1**	-0.014*	-0.018**	0.082***	0.128***	0.360***	0.163***	0.062***	0.005	-0.018**	1.000	
**Age**	-0.064***	-0.075***	-0.140***	-0.000	0.178***	0.314***	0.220***	0.077***	-0.163***	-0.048***	1.000

(*p<0.1, **p<0.05, ***p<0.01, represents significant at the 10%, 5% and 1% significance level.)

Table 3 presents the correlation matrix among the variables in this study. According to the data in Table 3, the correlation coefficient between the two measures of stock price collapse risk, Ncskew and Duvol, is 0.864, which is statistically significant at the 1% level. This indicates a strong positive correlation between these two indicators, supporting the rationale of using them as measures of stock price collapse risk.

### 5.3 Empirical results analysis

#### 5.3.1 Analyst optimism and stock price collapse risk

Before conducting the initial regression analysis, a Hausman test was performed to determine whether a fixed-effects model should be used. The regression results of analyst optimism and stock price collapse risk, controlling for other factors based on Model 5, are presented in Table 4. The column (1) displays the results where the Ncskew is used as the dependent variable for stock price collapse risk. The coefficient for analyst optimism is 0.041, which is statistically significant at the 10% level. In the column (2), the results are shown with the Duvol as the dependent variable for stock price collapse risk. The coefficient for analyst ratings is 0.016, which is statistically significant at the 10% level. These findings indicate a positive association between analyst optimism and stock price collapse risk, suggesting that higher extent of analyst optimism contribute to an increased occurrence of stock price collapse risk, thus confirming hypothesis 1a of this study. Additionally, in terms of the regression results with control variables, the coefficient for the debt-to-assets ratio (Lev) is negative and statistically significant at the 1% level, aligning with the conclusions drawn from the correlation analysis.

**Table 4:** Regression results of analyst ratings on stock price collapse risk.

Variable	(1)Ncskew	(2)Duvol
**Analyst**	0.041***	0.016*
	(2.66)	(1.85)
**Size**	0.010*	-0.004
	(1.70)	(-1.31)
**Lev**	-0.156***	-0.101***
	(-3.97)	(-4.51)
**Abord**	-0.066**	-0.032*
	(-2.23)	(-1.96)
**Flu**	-0.000	-0.000
	(-0.29)	(-0.08)
**CR1**	-0.088**	-0.056**
	(-2.19)	(-2.10)
**Age**	-0.052***	-0.034***
	(-2.75)	(-2.70)
**_Cons**	-0.147	0.131*
	(-1.01)	(1.68)
**Year**	Control	Control
**Industry**	Control	Control
**Observations**	18010	18010
**R-squared**	0.037	0.046

(*p<0.1, **p<0.05, ***p<0.01, represents significant at the 10%, 5% and 1% significance level; values in parentheses below represent the corresponding t-values.)

In order to explore how analyst optimism may impact stock price collapse risk through potential channels, considering the strong connection between institutional investors and securities analysts, this paper include institutional ownership as a variable to investigate its potential mediating effect. The findings of this analysis are presented in Table 5.

**Table 5:** Analyst rating and stock price collapse risk: The intermediary role of institutional shareholding.

Variable	(1)Ncskew	(2)Duvol	(3)Institution
**Analyst**	0.030***		
	(6.00)		
**Institution**		0.160***	0.101***
		(5.65)	(5.56)
**Size**	0.071***	-0.000	-0.011***
	(24.88)	(-0.02)	(-2.66)
**Lev**	-0.040**	-0.154***	-0.098***
	(-2.27)	(-3.93)	(-3.89)
**Abord**	0.110***	-0.086***	-0.044**
	(7.88)	(-2.88)	(-2.29)
**FLU**	-0.001	-0.000	0.000
	(-1.55)	(-0.26)	(0.01)
**CR1**	0.470***	-0.170***	-0.106***
	(22.63)	(-4.03)	(-3.91)
**Age**	0.053***	-0.062***	-0.040***
	(5.23)	(-3.30)	(-3.27)
**_cons**	-1.787***	0.264*	0.357***
	(-26.39)	(1.85)	(3.79)
**Year**	Control	Control	Control
**Industry**	Control	Control	Control
**Observations**	18010	18010	18010
**R-squared**	0.333	0.038	0.047

(*p<0.1, **p<0.05, ***p<0.01, represents significant at the 10%, 5% and 1% significance level; values in parentheses below represent the corresponding t-values.)

The provided table includes tests conducted in columns (1) and (2) to examine the relationship between analyst optimism and stock price collapse risk, yielding results consistent with those in Table 4. Furthermore, column (3) investigates the association between the mediating variable, institutional ownership (Institution), and the independent variable, analyst ratings (Analyst). The coefficient for analyst ratings (Analyst) is observed to be 0.030, significant at the 1% level. This indicates a positive correlation between analyst ratings and institutional ownership, suggesting that higher level of analyst optimism is linked to increased institutional ownership. These findings largely support the hypothesis that analysts may collude with institutional investors by issuing optimistic ratings to assist them in reducing their holdings in listed companies. As institutional ownership proportion rises, a deviation between stock prices and their actual values occurs. Consequently, there is an elevated risk of stock price collapse in the future, driven by the necessity for institutional reduction or the regression of stock price value. The validation of hypothesis 1b underscores the significance of this conclusion in terms of enhancing the regulation of the analyst industry and institutional investors by China’s securities exchanges. This proactive measure aims to prevent collusion of interests that could undermine the healthy development of the capital market.

#### 5.3.2 The moderating effect of information disclosure quality

To examine the moderating effect of information disclosure quality on the relationship between analyst optimism and stock price collapse risk, this study introduces an interaction term (Analyst*Score) between analyst ratings and information disclosure quality in Model 7. Table 6 presents the regression results of analyst ratings, information disclosure quality, and stock price collapse risk, controlling for other factors. The focus is primarily on investigating the impact of information disclosure quality on the relationship between analyst optimism and stock price collapse risk.

**Table 6:** The moderating effect of information disclosure quality.

Variable	(1)Ncskew	(2)Ncskew	(3)Duvol	(4)Duvol
**Analyst**	0.045***	0.055***	0.018*	0.022**
	(2.90)	(3.55)	(1.96)	(2.44)
**Score**	-0.032***	-0.032***	-0.015**	-0.015**
	(-3.31)	(-3.32)	(-2.39)	(-2.40)
Analyst*Score		0.069***		0.031**
		(2.98)		(2.27)
**SIZE**	0.015**	0.014**	-0.002	-0.002
	(2.48)	(2.38)	(-0.48)	(-0.54)
**LEV**	-0.183***	-0.185***	-0.113***	-0.114***
	(-4.59)	(-4.66)	(-4.36)	(-4.41)
**abord**	-0.062**	-0.060**	-0.030	-0.029
	(-2.09)	(-2.02)	(-1.58)	(-1.53)
**FLU**	-0.000	-0.000	-0.000	-0.000
	(-0.32)	(-0.24)	(-0.09)	(-0.04)
**CR1**	-0.075*	-0.075*	-0.050*	-0.050*
	(-1.86)	(-1.85)	(-1.92)	(-1.91)
**Age**	-0.053***	-0.053***	-0.034***	-0.034***
	(-2.83)	(-2.80)	(-2.82)	(-2.81)
**_cons**	-0.178	-0.222	0.117	0.097
	(-1.23)	(-1.55)	(1.24)	(1.03)
**Year**	Control	Control	Control	Control
**Industry**	Control	Control	Control	Control
***N***	18010	18010	18010	18010
***R*** ^**2**^	0.038	0.038	0.046	0.046

(*p<0.1, **p<0.05, ***p<0.01, represents significant at the 10%, 5% and 1% significance level; values in parentheses below represent the corresponding t-values.)

In relation to the primary variables, the coefficients of the interaction term (Analyst*Score) between analyst optimism and information disclosure quality, after being centered, are found to be 0.069 and 0.031 for the Ncskew and the Duvol, respectively, significance at the 1% and 5% levels, respectively. These results suggest that improving information disclosure quality enhances the positive influence of analyst optimism on stock price collapse risk. It also indicates the presence of a self-adjustment and correction mechanism in the Chinese securities market to address excessively optimistic analyst ratings. As the information disclosure quality of listed companies improves, this mechanism becomes more effective in rectifying overvalued companies by driving their stock prices downward, potentially leading to a collapse that aligns with their intrinsic value. These findings provide empirical support for the study’s theoretical framework and validate hypothesis 2b. Moreover, they offer theoretical guidance for enhancing regulatory measures pertaining to information disclosure quality by Chinese exchanges. From a practical standpoint, these findings have significant implications in guiding analysts to form realistic growth expectations for listed companies in the Chinese securities market.

Regarding analyst ratings (Analyst), the coefficients between analyst ratings and the Ncskew and the Duvol are estimated to be 0.055 and 0.022, respectively, significance at the 1% and 5% levels, respectively. These results confirm the presence of a substantial positive correlation between analyst optimism and stock price collapse risk, further corroborating hypothesis 1a of this study. Additionally, the relationships between the control variables, such as the Debt-to-asset ratio (Lev), board size (Abord), the ownership percentage of the largest shareholder (CR1), and company age (Age), and stock price collapse risk align with the previous correlation analysis and the regression results obtained from Model 5.

#### 5.3.3 Heterogeneity test

Looking at the situation objectively, there could be some inherent bias in the initial regression analysis that examines the relationship between analyst optimism and the risk of stock price collapse. A notable concern is the presence of heterogeneity in the characteristics of different companies, which might mask the variations in analyst evaluations and their impact on stock price collapse risk. To tackle this issue, the study conducts a heterogeneity test by dividing the sample firms into two categories: state-owned enterprises and non-state-owned enterprises, based on their specific attributes. The results of the heterogeneity test are presented in Table 7.

**Table 7:** Analyst rating and risk of stock price collapse: Heterogeneity test.

	state-owned enterprises	non-state-owned enterprises	state-owned enterprises	non-state-owned enterprises
	(1)	(2)	(3)	(4)
	Ncskew	Ncskew	Duvol	Duvol
**Analyst**	0.060**	0.023	0.026*	0.006
	(2.28)	(1.20)	(1.68)	(0.55)
**SIZE**	0.018*	0.017**	0.000	-0.002
	(1.90)	(2.03)	(0.05)	(-0.43)
**LEV**	-0.191***	-0.093*	-0.104**	-0.069**
	(-2.64)	(-1.93)	(-2.23)	(-2.22)
**abord**	-0.093*	-0.019	-0.059*	-0.003
	(-1.81)	(-0.53)	(-1.76)	(-0.14)
**FLU**	-0.002	0.000	-0.003	0.000
	(-0.34)	(0.23)	(-0.57)	(0.44)
**CR1**	-0.012	-0.073	-0.010	-0.058*
	(-0.16)	(-1.48)	(-0.20)	(-1.80)
**Age**	-0.081*	-0.016	-0.065**	-0.009
	(-1.89)	(-0.75)	(-2.32)	(-0.66)
**_cons**	-0.209	-0.452**	0.128	-0.002
	(-0.79)	(-2.29)	(0.73)	(-0.01)
**Year**	Control	Control	Control	Control
**Industry**	Control	Control	Control	Control
***N***	5196	12814	5196	12814
***R*** ^**2**^	0.058	0.031	0.062	0.041

(*p<0.1, **p<0.05, ***p<0.01, represents significant at the 10%, 5% and 1% significance level; values in parentheses below represent the corresponding t-values.)

The analysis of the differences between state-owned enterprises and non-state-owned enterprises reveals results as shown in Table 7. It is found that analyst optimism do not significantly influence the stock price collapse risk of non-state-owned enterprises. However, in the case of state-owned enterprises, the regression analysis using the Ncskew as the dependent variable shows a significant coefficient of 0.060 for analyst optimism at the 5% level. Similarly, when the Duvol is used as the dependent variable, the coefficient of analyst ratings is 0.026, significant at the 10% level. These results indicate a significant positive impact of analyst optimism on the stock price collapse risk of state-owned enterprises.

This finding is attributed to the distinctive characteristics of state-owned enterprises, which exhibit a certain aversion to risk. Analysts, in response, tend to provide overly optimistic ratings for this type of company. Consequently, when the actual performance of a company does not align with the optimistic ratings provided by analysts, such companies are more susceptible to experiencing a stock price collapse.

### 5.4 Endogeneity and robustness tests

#### 5.4.1 Endogeneity test

Considering the potential endogeneity issue in estimating Model 5, which suggests that certain characteristics of listed companies are related to their stock price collapse risk, and simultaneously, these characteristics may also influence analyst ratings, it is important to examine the robustness of the results and demonstrate that the dependent variable, stock price collapse, does not have a reverse impact on the independent variable (analyst optimism). To address these concerns, this study employs a System GMM model for endogeneity testing. The System GMM regression method is a panel data analysis approach that, compared to the Fixed Effects Model, offers several advantages:

Firstly, it effectively addresses endogeneity issues, particularly those arising in panel data. By using lagged variables and differenced variables as instrumental variables, the System GMM regression method estimates regression coefficients, thereby reducing estimation bias caused by endogeneity. Secondly, it leverages more time dimensions, including lagged variables and differenced variables, to estimate regression coefficients. This allows for better capturing of time-related variations and dynamic features, particularly suitable for addressing time-related issues in panel data. Thirdly, it provides higher efficiency compared to the Fixed Effects Model. By simultaneously using lagged variables and differenced variables to estimate regression coefficients, it maximizes the use of available information, reducing estimation variance.

However, there are differences between the System GMM regression method and the Fixed Effects Model:

Firstly, in handling heterogeneity issues, System GMM regression is relatively weaker compared to the Fixed Effects Model. Although System GMM regression can control individual heterogeneity by introducing individual fixed effects, complete elimination of individual heterogeneity in panel data is often challenging. Secondly, System GMM regression has relatively higher data requirements, necessitating longer time dimensions and larger sample sizes for reliable estimation results. In contrast, the Fixed Effects Model has lower data requirements and is suitable for smaller sample sizes and shorter time dimensions.

The results are presented in Table 8:

**Table 8:** Regression results of dynamic panel model under system GMM model.

	(1)	(2)
**L.Ncskew**	-0.224*	
	(-1.68)	
**L.Duvol**		-0.204*
		(-1.75)
**Analyst**	0.538***	0.414***
	(4.70)	(4.63)
**Size**	-0.054***	-0.040***
	(-3.24)	(-3.67)
**Lev**	-0.074	-0.047
	(-1.08)	(-1.16)
**Abord**	0.034	0.020
	(0.75)	(0.71)
**FLU**	-0.002	-0.001
	(-0.96)	(-0.49)
**CR1**	0.376	0.276
	(0.86)	(0.95)
**Age**	-0.179***	-0.098***
	(-5.41)	(-3.99)
**_cons**	-1.101***	-0.984**
	(-2.66)	(-2.40)
**AR(1)**	0.009	0.002
**AR(2)**	0.110	0.125
**Sargan-test**	0.128	0.302

(*p<0.1,**p<0.05, ***p<0.01, represents significant at the 10%, 5% and 1% significance level; values in parentheses below represent the corresponding t-values.)

In Table 8, the Sargan test is performed to assess the validity of the instrumental variables and determine if there is an issue of over-identification. The null hypothesis in the Sargan test assumes that the instrumental variables are valid. The p-values obtained from the Sargan test, all exceeding 0.05, indicate that the instrumental variables used in this study do not suffer from over-identification problems. Therefore, the instrumental variables are considered valid.

The Arellano-Bond test includes two variations: AR (1) and AR (2), which examine the presence of first-order and second-order serial correlation in the differenced residuals of the model. The null hypothesis assumes the absence of autocorrelation. Generally, if the differenced residuals do not exhibit second-order autocorrelation, the GMM model is considered valid. In the regression results, the p-values for the AR (2) test are 0.110 and 0.125, respectively, both greater than 0.05. This suggests that there is no second-order autocorrelation in the residuals, providing further evidence for the validity of the model.

#### 5.4.2 Robustness test of analyst optimism and stock price collapse risk

In this study, the market-specific data of the coefficient of negative return skewness (Ncskew) and the stock price volatility ratio of weekly returns (Duvol), denoted as Ncskew_Mdtl and Duvol_Mdtl, were used as substitutes for the original data on stock price collapse risk. These variables were incorporated into the original model for regression analysis. The regression results are presented in Table 9:

**Table 9:** The robustness test of analyst rating on the risk of stock price collapse.

Variable	(1)Ncskew	(2)Duvol
**Analyst**	0.039**	0.015*
	(2.46)	(1.81)
**Size**	0.025***	0.010***
	(4.12)	(2.70)
**Lev**	-0.091**	-0.045*
	(-2.19)	(-1.71)
**Abord**	-0.032	-0.019
	(-1.05)	(-0.98)
**Flu**	-0.000	0.000
	(-0.21)	(0.10)
**CR1**	-0.038	-0.022
	(-0.91)	(-0.83)
**Age**	-0.020	-0.004
	(-1.05)	(-0.41)
**_cons**	-0.717***	-0.363***
	(-4.69)	(-5.03)
**Year**	Control	Control
**Industry**	Control	Control
**Observations**	18010	18010
**R-squared**	0.034	0.039

(*p<0.1, **p<0.05, ***p<0.01, represents significant at the 10%, 5% and 1% significance level; values in parentheses below represent the corresponding t-values.)

The regression results in Table 9 reveal that when Ncskew is used as the dependent variable, there is a significant positive correlation between analyst ratings and the coefficient of 0.039 at a 5% level of significance. This implies that higher analyst ratings are linked to greater negative skewness of returns, indicating a higher probability of future stock price collapse risk. Similarly, in the regression with Duvol as the dependent variable, there is a significant positive correlation between analyst ratings and a coefficient of 0.015 at a 10% level of significance. This suggests that higher analyst ratings are associated with increased volatility in stock returns, which implies a higher likelihood of future stock price collapse risk for listed companies. These findings are consistent with the regression results obtained from Model 5 with minor variations. Additionally, the conclusions regarding the control variables are largely in line with those derived from Model 5.

#### 5.4.3 Robustness test of analyst optimism, information disclosure quality, and stock price collapse risk

To ensure the robustness of the results, this study replaces the original data on stock price collapse risk with market-specific data on the Ncskew and the Duvol. These variables, namely Ncskew_Mdtl and Duvol_Mdtl, are used as substitutes in the regression analysis. The regression results are presented in Table 10:

**Table 10:** Analyst rating, quality of information disclosure and robustness test of stock price collapse risk.

Variable	(1)Ncskew	(1)Ncskew	(2)Duvol	(2)Duvol
**Analyst**	0.044***	0.054***	0.018*	0.021***
	(2.80)	(3.43)	(1.87)	(3.23)
**Score**	-0.046***	-0.046***	-0.022***	-0.022***
	(-4.50)	(-4.52)	(-3.45)	(-6.83)
Analyst*Score		0.067***		0.022*
		(2.70)		(1.83)
**Size**	0.032***	0.031***	0.013***	0.013*
	(5.12)	(5.02)	(3.23)	(2.07)
**Lev**	-0.129***	-0.131***	-0.063**	-0.064**
	(-3.06)	(-3.13)	(-2.31)	(-2.36)
**Abord**	-0.027	-0.025	-0.016	-0.015
	(-0.87)	(-0.80)	(-0.81)	(-1.29)
**Flu**	-0.000	-0.000	0.000	0.000
	(-0.25)	(-0.18)	(0.06)	(0.13)
**CR1**	-0.019	-0.019	-0.013	-0.013
	(-0.46)	(-0.45)	(-0.48)	(-0.63)
**Age**	-0.023	-0.022	-0.005	-0.005
	(-1.17)	(-1.14)	(-0.42)	(-0.54)
**_cons**	-0.761***	-0.804***	-0.384***	-0.399***
	(-5.00)	(-5.37)	(-3.91)	(-3.14)
**Year**	Control	Control	Control	Control
**Industry**	Control	Control	Control	Control
**Observations**	18010	18010	18010	18010
**R-squared**	0.035	0.035	0.040	0.040

(*p<0.1, **p<0.05, ***p<0.01, represents significant at the 10%, 5% and 1% significance level; values in parentheses below represent the corresponding t-values.)

Based on the regression findings mentioned above, it can be observed that the coefficients of analyst ratings and information disclosure quality, along with the interaction term (Analyst*Score) after being centered, are 0.067 and 0.022, respectively, significant at the 1% and 10% levels, respectively. These results indicate that the positive impact of analyst ratings on Ncskew and the Duvol is strengthened by information disclosure quality. This suggests that as the information disclosure quality of listed companies improves, enhancing the transparency of corporate information and enabling analysts to access higher-quality public information to enhance the accuracy of their research reports, it will further reinforce the self-adjustment and correction mechanism of securities market towards companies with excessively optimistic valuations. Consequently, this mechanism may lead to downward adjustments in the stock prices of such companies, potentially resulting in stock price collapse, to achieve the purpose of correction. These conclusions align with the regression results obtained from Model 7 and do not exhibit significant variations. Furthermore, the conclusions regarding the control variables are also generally consistent with those obtained from Model 7.

## 6. Conclusion and recommendations

### 6.1 Research conclusions

This study utilized A-share listed companies on the Shanghai and Shenzhen stock exchanges from 2011 to 2020 as the research sample to investigate the impact of analyst optimism and information disclosure quality on stock price collapse risk. Additionally, the study examined the transmission path of analyst optimism on stock price collapse risk. Through theoretical analysis and empirical research, the following conclusions have been drawn:

1. The evident strong positive correlation between analyst optimism and the risk of stock price collapse underscores the intricate dynamics within the securities market. Despite analysts playing a crucial role as information intermediaries, their optimistic reports contribute to the challenge of mitigating information asymmetry. In a market where accurate and timely information dissemination is paramount, conflicts of interest and cognitive biases hinder the effectiveness of analysts in fulfilling this role. Upon deeper exploration of this relationship, it becomes apparent that the prevalence of analyst optimism not only hinders the efficient operation of the capital market but also deepens information asymmetry. The inclination toward overly optimistic reports from analysts raises concerns about the transparency and accuracy of information available to investors. This, in turn, exacerbates the challenges investors face in making well-informed decisions. In the context of stakeholder interests, the inclination of analysts toward optimism may inadvertently assist stakeholders in concealing negative information. This potential alignment of interests could contribute to a scenario where crucial information is not adequately reflected in market assessments, creating an environment susceptible to future stock price collapses. Furthermore, the appeal of optimistic reports to small and medium-sized investors may lead to a herd effect, where investors follow optimistic recommendations without fully assessing the associated risks. In conclusion, understanding and addressing the impact of analyst optimism on stock price collapse risk is crucial for fostering a more transparent and efficient capital market. Recognizing the challenges posed by conflicts of interest and cognitive biases in analyst reports opens the door for potential regulatory interventions and industry practices that promote unbiased and accurate information dissemination, ultimately safeguarding the interests of all market participants.
2. The notable positive correlation between the interaction term of analyst optimism and information disclosure quality and the risk of stock price collapse highlights a nuanced dynamic within the A-share market. Specifically, when listed companies exhibit high levels of information disclosure quality, the favorable influence of analyst optimism on stock price collapse risk becomes more pronounced. This intricate relationship underscores the potential regulatory role of the "information disclosure assessment" system in the A-share market. The findings suggest that the A-share market’s "information disclosure assessment" system can act as a regulatory mechanism. It has the capacity to automatically adjust the stock prices of companies influenced by overly optimistic growth expectations from analysts. This automatic correction mechanism serves to rectify valuations, potentially averting stock price collapses and facilitating a more rational and reflective pricing regression. In essence, the observed correlation emphasizes the intricate interplay between analyst optimism, information disclosure quality, and stock price collapse risk. Leveraging this understanding, regulators can explore and refine existing systems to create a more resilient and responsive market environment, ensuring that stock valuations align more closely with fundamental realities and minimizing the risk of abrupt price collapses.
3. The influence of analyst optimism on stock price collapse extends through the intricate mechanism of institutional ownership. The observed trend indicates that as analyst ratings experience an upward trajectory, there is a corresponding increase in institutional ownership. This dynamic interplay between analyst optimism and institutional ownership contributes to an increased likelihood of future stock price collapses. The correlation between rising analyst ratings and increased institutional ownership adds a noteworthy aspect to the risk assessment landscape. It suggests that the impact of analyst optimism extends beyond individual investor decisions and permeates into the actions of institutional stakeholders. As institutional ownership intensifies in response to optimistic analyst ratings, the collective weight of these influential investors may inadvertently contribute to an increased risk of subsequent stock price collapses. In essence, this observation underscores the interconnected nature of market dynamics. Analyst optimism not only influences individual investor behavior but also has a ripple effect on institutional decisions, creating a potential alignment in actions that can amplify the risk of future stock price collapses. Recognizing and understanding this linkage can assist market participants, regulators, and institutional investors in refining strategies and risk management approaches to foster a more resilient and stable market environment.

### 6.2 Comparison with previous studies

Presently, the academic community has extensively studied the relationship between analysts and stock price collapse. Comparing the findings of this study with previous research, it is evident that most studies emphasize the impact of analysts’ subjective or irrational analyses on the market, potentially increasing the risk of stock price collapse. In these studies, internal corporate governance is considered a crucial aspect that requires close monitoring. Simultaneously, these studies generally assert that avoiding information asymmetry is of paramount importance, particularly the timely communication of negative news to the market to prevent the accumulation of negative information, which could trigger a stock price collapse. However, compared to previous research, few scholars have considered the role of institutional ownership in this relationship. Institutional investors play a pivotal role in the market, and their behavior can significantly impact stock price collapse. This is an innovation in this study, as we delve into the more detailed aspects of market dynamics by exploring the interaction between analysts’ optimism and institutional ownership. Additionally, this study introduces information disclosure quality as a moderating factor. In previous research, few scholars have considered information disclosure quality, but this study argues that improving information disclosure quality can mitigate the impact of analysts’ optimism on the risk of stock price collapse to some extent. The introduction of this perspective injects new elements into the current research field and deepens our understanding of the relationship between analyst behavior and market stability. Therefore, the uniqueness of this study lies in the comprehensive consideration of institutional ownership and information disclosure quality, as well as the in-depth exploration of their interplay with analysts’ optimism, offering a more comprehensive and profound analytical framework for the academic community.

### 6.3 Recommendations

Based on the research conclusions of this study, the following recommendations are proposed:

1. For Regulatory authorities: Regulatory authorities should refine industry guidelines and concurrently explore a more rational system for assessing analysts and implementing reward-penalty mechanisms. Diligent analysts should receive a series of rewards, while non-diligent analysts should face a series of punitive measures. This approach aims to promote regulatory compliance within the analyst industry, enhance the quality of analysts’ predictions, and enable them to better fulfill their role as information intermediaries. Simultaneously, for listed companies with a good rating for information disclosure quality, the exchange can provide certification services for the authenticity of their announcements. In contrast, companies with suboptimal information disclosure quality ratings should be warned when communicating with investors and offered relevant support services, guiding them toward improving their information disclosure practices in the future.
2. For securities analysts: The advantages within the analyst industry empower analysts to acquire more comprehensive fundamental information about listed companies compared to ordinary investors, enabling them to conduct more professional analyses of corporate operations. In the process of releasing research reports, analysts should adhere to professional ethics, value their reputation, base their analyses on facts, and present analytical conclusions thoroughly in research reports, promptly conveying them to the market and investors. The analyst community should actively establish a positive image before investors and, concurrently, fulfill their role as external supervisors of listed companies. This ensures that listed companies can develop while safeguarding the interests of all parties. Simultaneously, the analyst industry should enhance self-disciplinary supervision and effectively monitor analyst behavior.
3. For listed companies: Listed companies, while focusing on daily production and operational activities to enhance corporate performance, should also fully utilize existing channels of information disclosure. It is crucial to proactively disclose the company’s operational status to the market in accordance with securities regulations. Furthermore, companies should strengthen their connections with analysts to assist them in making reasonable and accurate forecasts of the company’s performance, thus providing correct guidance to the market’s expectations. In addition, listed companies should enhance internal governance and personnel training, implement reasonable equity incentives for key employees, and, in the event of negative information arising during the company’s operations, promptly release risk warnings to the market instead of concealing negative news. This ensures that performance risks can be promptly addressed, preventing the accumulation of negative information that could lead to a stock price collapse.

### 6.4 Limitations and future research directions

#### 6.4.1 Limitations of the study

In the scrutiny of existing research, it is crucial to identify and acknowledge potential limitations. Firstly, there is a limitation in the sample: The current study may have limitations in sample size, especially concerning data sources and the coverage of companies. Future research efforts should focus on expanding the sample size and diversity to enhance the generalizability and reliability of the findings. Secondly, methodological challenges exist: Some research methods may face challenges, such as the complexity of analyzing analyst optimism and stock price collapse risk. Future research could explore more advanced methods and techniques to improve the methodological rigor and accuracy of the research.

#### 6.4.2 Future research directions

Building upon an in-depth understanding of the current research limitations, future studies could explore the following directions:

1. Impact of Analyst Optimism: The influence of analyst optimism on stock prices has been a subject of continuous interest. Future research can delve into the motivations and reasons behind analyst optimism, as well as its effects on investor behavior and market volatility. Further studies could propose new methods and models to measure the accuracy and bias of analyst predictions and explore the long-term effects of optimistic estimates on the market.
2. Enhancement of Information Disclosure: Information disclosure plays a crucial role in maintaining market transparency and investor interests. Future research could focus on improving the quality and accuracy of information disclosure and enhancing its comprehensibility to investors. Additionally, researchers could investigate how emerging technologies, such as artificial intelligence and big data analytics, can improve the efficiency and timeliness of information disclosure.
3. Prediction and Management of Stock Price Collapse Risk: Stock price collapse risk has significant implications for investors and market stability. Future research could attempt to develop new models and indicators for predicting the risk of stock price collapse and propose corresponding risk management strategies. Studies could also focus on the causes and mechanisms of stock price collapse to better understand the impact of market irrationality and investor sentiment on stock prices.

## Supporting information

S1 Data (XLS)
